# The Development of Controllable Magnetic Driven Microphysiological System

**DOI:** 10.3389/fcell.2019.00275

**Published:** 2019-11-07

**Authors:** Jia-Wei Yang, Yu-Wei Chen, Pei-Yi Ho, Liane Jiang, Kuan Yu Hsieh, Sheng-Jen Cheng, Ko-Chih Lin, Huai-En Lu, Hsien-Yi Chiu, Shien-Fong Lin, Guan-Yu Chen

**Affiliations:** ^1^Department of Electrical and Computer Engineering, College of Electrical and Computer Engineering, National Chiao Tung University, Hsinchu, Taiwan; ^2^Institute of Biomedical Engineering, College of Electrical and Computer Engineering, National Chiao Tung University, Hsinchu, Taiwan; ^3^Centre for Ophthalmology, Section for Experimental Ophthalmic Surgery and Refractive Surgery, University of Stuttgart, Stuttgart, Germany; ^4^Bioresource Collection and Research Center, Food Industry Research and Development Institute, Hsinchu, Taiwan; ^5^Department of Dermatology, National Taiwan University Hospital Hsin-Chu Branch, Hsinchu, Taiwan; ^6^Department of Dermatology, College of Medicine, National Taiwan University, Taipei, Taiwan; ^7^Department of Dermatology, National Taiwan University Hospital, Taipei, Taiwan; ^8^Department of Biological Science and Technology, National Chiao Tung University, Hsinchu, Taiwan

**Keywords:** magnetic driven, microphysiological systems, human alveolar epithelial cells, barrier function, silica nanoparticles

## Abstract

Current research has enabled the use of microphysiological systems and creation of models for alveolar and pulmonary diseases. However, bottlenecks remain in terms of medium- and long-term regulation of cell cultures and their functions in microchannel systems, as well as in the enhancement of *in vitro* representation of alveolar models and reference values of the data. Currently used systems also require on-chip manufacturing of complex units, such as pumps, tubes, and other cumbersome structures for maintaining cells in culture. In addition, system simplification and minimization of all external and human factors major challenges facing the establishment of *in vitro* alveolar models. In this study, a magnetically driven dynamic alveolus cell-culture system has been developed to use controlled magnetic force to drive a magnetic film on the chip, thereby directing the fluid within it to produce a circulating flow. The system has been confirmed to be conducive with regard to facilitating uniform attachment of human alveolar epithelial cells and long-term culture. The cell structure has been recapitulated, and differentiation functions have been maintained. Subsequently, reactions between silica nanoparticles and human alveolar epithelial cells have been used to validate the effects and advantages of the proposed dynamic chip-based system compared to a static environment. The innovative concept of use of a magnetic drive has been successfully employed in this study to create a simple and controllable yet dynamic alveolus cell-culture system to realize its functions and advantages with regard to *in vitro* tissue construction.

## Introduction

The physiological and biological functions of alveolar epithelial type II (AEII) cells involve synthesizing, secreting, and recycling of all surfactant components to regulate surface tension within the entire alveolus (Fehrenbach, 2001; Mason, 2006). Although AEII cells only account for approximately 5% of the total alveolar surface area, they comprise 60% of the total number of alveolar cells (Crapo et al., 1982; Fehrenbach, 2001). They therefore play an essential role in maintaining the overall function of alveoli, thereby serving as progenitors of alveolar epithelial cells (Barkauskas et al., 2013; Hogan, 2018; Nabhan et al., 2018; Zacharias et al., 2018). Although isolation techniques and collection pipelines for primary alveolar cells have been well established, primary AEII cells tend to differentiate into AEI cells when employing common dish-culture methods (Fujino et al., 2012; Nikolic et al., 2017; Zheng et al., 2017). In addition, many studies have confirmed the difficulty involved in maintaining AEII cells with surfactant producing functions using *in vitro* culture methods (Mao et al., 2015). Consequently, cell-growth and differentiation regulation, representativeness enhancement of *in vitro* alveolar models, enhancement of reference data values, and complete recapitulation of functions of the human alveolus have become important research topics in recent times (Bove et al., 2014; Jain et al., 2015; Mao et al., 2015).

Cell culture techniques employing microfluidic devices have become increasingly popular, and microfluidic systems, over the years, have realized versatility, miniaturization, and automation of biochemical processes (Halldorsson et al., 2015; Chiu et al., 2017). Flexibly designing a chip device for each experiment or cell, using different parameters during each experiment to improve operational flexibility, performing perfusion cell culture, and reducing reagent consumption have all become possible (Mark et al., 2010; Byun et al., 2014). Extant researchers have indicated that alveolar epithelial cells can be cultured on a film placed on a microchip, and differences in gas pressures can be used to simulate the respiratory motion of the human body (Huh et al., 2010; Stucki et al., 2015a; Guenat and Berthiaume, 2018). It has also been determined that differences exist in the molecular penetration rate, wherein a higher penetration rate can be observed in dynamic environments compared to static environments (Huh et al., 2012; Esch et al., 2015). The secretion of cytokine is also much higher in dynamic environments compared to that in static environments (Stucki et al., 2015b). Researchers have also investigated the culture of AEII cells in dynamic systems and observed that differences in the flow rate greatly affect cell morphology and activity as well as proteins secreted by them (Grek et al., 2009; Stucki et al., 2018). Different results have been observed for different cells, albeit at the same flow rate, and it has been observed that quick and convenient control of dynamic culture environment parameters and the corresponding flow rate greatly assist the functioning of alveolar epithelial cells while also facilitating microenvironment construction (Douville et al., 2011; Freund et al., 2012).

In current microfluidics fluid-control practices, however, the principal driving method involves driving the medium through a syringe or peristaltic pump to provide a state of stable laminar flow inside on-chip channels. The main purpose of this state is to simulate fluid flow within human body tissues, thereby providing a fluidic environment that simulates the human body (Tehranirokh et al., 2013; Kim et al., 2014; Kimura et al., 2018). For example, in the recently developed lung modeling technology, alveolar cells are constructed on a chip with the objective of replacing studies involving animal subjects while also generating more accurate and reliable preclinical data (Benam et al., 2015, 2016; Ronaldson-Bouchard and Vunjak-Novakovic, 2018). However, creation of such a pump system requires many tubes to be interconnected, and a syringe and many converters are required to connect the chip with a medium reservoir (Temiz et al., 2015). Channel pretreatment is also complicated, requiring high-temperature sterilization. In addition, peristaltic pumps are to be squeezed, thereby resulting in brittleness and instability of channels. Further, the more the channels are used, the greater is the difference between the theoretical and actual flow rates on a chip. Such a system often requires use of numerous auxiliary devices, and therefore, placement of these auxiliary components to minimize the effect of all external and human factors currently constitutes a major challenge facing the development of the technology pertaining to alveolar cell culture on microfluidic systems.

**SCHEME 1:**
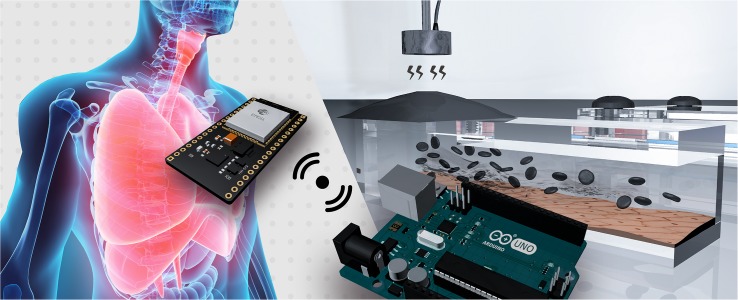
The magnetically driven dynamic alveolus cell-culture system facilitates selection of magnetically controllable output modes, and employs a magnetic film as a medium to control fluid circulation through on-chip microchannel.

To enhance the *in vitro* physiological function of alveolar cells, the design of a physical environment capable of simulating real life conditions is crucial to ensure that cell growth occurs under culture conditions that are as close as possible to *in vivo* conditions in terms of spatial parameters. In this study, a magnetically driven alveolar cell chip-based system has been developed equipped with a magnetic film. Integration of the thin magnetic film and controllable magnetic output with a microchannel system provides a simple and controllable fluid dynamic environment that recapitulates cell structures, physical and chemical microenvironments, and biomimetic nature of fluid flow. It also offers advantages pertaining to *in vitro* analysis of tissue functions and alveolar environmental activity (Scheme 1). This system is also expected to realize the advantages of device simplification by way of removal of complicated structures, such as pumps and tubes. Moreover, nanoparticles were introduced and the toxicological effects were compared between dynamic and static environments via use of the proposed alveolar system, thereby validating its potential for use in future bionic engineering applications concerning tissue and organ chips.

## Materials and Methods

### Design of Proposed Magnetic Driven Chip

The PDMS material used in this study contained Sylgard 184A base and Sylgard 184B curing agent. In this study, the PDMS chips were prepared at base-to-curing agent ratios of 10:1 to 15:1 for use in subsequent experiments (Dardik et al., 2005). The overall structure of the proposed magnetic driven chip can be primarily divided into two parts – (i) a PDMS-based three-layer chip comprising a top layer, middle layer (microchannel), and glass slide (bottom layer) and (ii) a chip fixture comprising an upper cover and mounting base. The layers in the first part are bonded to each other by oxygen-plasma-activated bonding. The upper layer of the chip is a rectangular cuboid measuring 75 mm (L) × 25 mm (W) × 5 mm (H) and contains square slots measuring 20 mm (L) × 20 mm (W) × 5 mm (H) and 5 mm (L) × 5 mm (W) × 5 mm (H). These empty slots are primarily used to hold the cell medium. The second layer is a 55 mm (L) × 1.5 mm (W) × 0.2 mm (H) microchannel layer while the third layer is a 75 mm (L) × 25 mm (W) × 1 mm (H) glass slide. After the second and third layers are bonded, a microchannel measuring 55 mm (L) × 1.5 mm (W) × 0.2 mm (H) is formed to constitute the area for cell culture. The upper cover of the second part measures 86 mm (L) × 36 mm (W) × 2 mm (H) in outer dimensions, and its periphery contains an M6 hole (a hole with diameter measuring 6 mm and containing a thread). In addition, two square areas [measuring 19 mm (L) × 19 mm (W) and 7 mm (L) × 7 mm (W)] are carved of the inside. Likewise, the base has external dimensions of 86 mm (L) × 36 mm (L) × 8 mm (W), and its periphery contains an M6 hole.

### Magnetic Film Preparation

The CI-powder (44890, Sigma–Aldrich) used in this study had a particle size distribution of 5–9 μm, a density of 7.86 g/mL, and a purity of 99.5%, with a color that was pale to dark gray. The magnetic film was prepared by blending a specific ratio of PDMS solution (a 15:1 ratio of Sylgard 184A base to Sylgard 184B curing agent) (Royet et al., 2017). PDMS solution was next added at CI-powder-to-PDMS weight ratios of 2:1, 1:1, 2:1, and 4:1 (CI:PDMS), and was stirred thoroughly for 10 min until uniform. The CI–PDMS mixture was then poured into the mold, and the membrane containing CI–PDMS was placed in a vacuum plate for 10 min to remove air from the CI–PDMS. It was next placed in an oven at 80°C for 2 h for curing, and the preparation of the magnetic film was completed. The magnetic film was next washed with 70% alcohol, and the remaining liquid was removed using nitrogen gas.

### Electromagnet System and Support Bracket

Mechanical 3D computer-aided design software (Solidworks, 2015, Concord, MA, United States) was used to draw the system support bracket, which primarily consisted of a rectangular cuboid with dimensions of 100 mm (L) × 50 mm (W) × 35 mm (H). The left side had a rectangular slot of 75 mm (L) × 27 mm (W) × 30 mm (H) where the magnetic driven chip placed, and an area on top was designed to allow the electromagnet to be mounted above the chip. A 3D printer was then used to output the completed object. The constant current LED driver module (LuxDrive BuckPuck 3021, Randolph, VT, United States) contained six pins: high output voltage DC (LED+), low output voltage DC (LED−), control pin voltage (CTL), and reference regulator current (REF), high input voltage DC (VIN+), and low input voltage DC (VIN−). The suction-cup electromagnet (HCNE1-P30/22) was wired by connecting to the output voltage DC of driver module. The input voltage DC was connected to the DC 12V switching power supply adapter (APV-35-12, Mean Well Enterprises Co., Ltd.). After the CTL was connected to the pin specified by the code of the Arduino, the signal was transmitted to the driver module and run according to the logic of the code.

### Code Writing and Control

The CTL pins of driver module were connected to the PIN_3, 5, 6, 9, 10, and 11 (pulse width modulation, PWM) on the Arduino UNO R3 development board, to complete pre-operation (Badamasi, 2014). For program coding, the pins were first arranged in a void setup(), and the pin was set to OUTPUT. Next, two loop function were written in the void loop() to control the increase and decrease of the magnetic force. The first loop was for controlling the increase in magnetic force. In the first loop, the variable *a* was set to a value of 0 and incremented by 1 after each loop. In addition, the delay() syntax was used to control the time. The loop terminated when the variable *a* equaled 255. The second loop was then executed using the same principle in reverse to control the decrease in magnetic force, thus completing the coding of the magnetic system.

### Measurement of Magnetic Film Deformation

The electromagnet was placed under the magnetic film, and then the electromagnet was opened to deform the film. The deformation of the film was recorded using a 3D shape measuring instrument (S neox non-contact 3D optical profiler, Sensofar). An image of the magnetic film as well as the 3D reconstructed image and film deformation were obtained directly from the measurement results. In addition, the deformation values were directly measured and the data were plotted using OriginLab and GraphPad Prism software.

### Measurements of Flow Rate in a Microchannel

The 2 mL of polystyrene particles having a particle size of 19.2 μm and a concentration of 2 × 10^5^ beads/mL were injected into the magnetic driven chip. The chip was then placed on the Electromagnet System, and Arduino was used to program the magnetic regulation of the particle flow rate in the microchannels. The flow rate measurement was divided into sinusoidal and stepped sinusoidal signals cyclic flows. First, a sinusoidal function generator was used to generate 8 bits (0–255) for a total of 256 values. Next, a matrix with a capacity of 256 values was coded in Arduino, and then the same loop was used to iterate the initial value from 0 to 255. Thus, a complete sine wave signal could be obtained, and the rate of flow could be adjusted by delaying the time of each iteration. Finally, a microscope was used to observe the flow of particles in the microchannels, and video was captured using the recording mode of the microscope software at 60 fps. The flow velocity (μm/s) of the particles was inferred from the recorded video using the tracker video analysis and modeling tool^1^. After the flow velocity (μm/s) was determined and then converted to the volumetric flow rate (μL/min) in the microchannels in different modes for continuous cell culture.

### Cell Culture and Analysis

The human pulmonary alveolar epithelial cells (HPAEpiC) used in this study were obtained from ScienCell Research Laboratories (Catalog #3200). First, 2 μg/cm^2^ poly-L-lysine (Sigma–Aldrich) was added to a T-75 flask (75 cm^2^) before inoculation of the cells. Next, cells were inoculated at a density of 10,000–15,000 cells/cm^2^ into the culture dish with alveolar epithelial cell medium (AEpiCM, ScienCell), epithelial cell growth supplement (EpiCGS, ScienCell), fetal bovine serum (FBS, ScienCell), and penicillin/streptomycin solution (P/S, ScienCell), and then cultured at 37°C and 5% CO_2_. The culture medium was changed every other day, and then every 3 days until the confluent state was reached. In subsequent analytical experiments, live and dead cells were detected by staining with green and red fluorescence, respectively, using a LIVE/DEAD assay (GeneCopoeia^TM^), and cell viability was verified by fluorescence microscopy. Next, we added the primary antibody SPC (AB3786, Millipore) at a concentration of 1:1000, a Cy3-fluorescence labeled goat anti-rabbit secondary antibody (2707846, Cell signal) at a concentration of 1:200, and Alexa Fluor^®^ 488-labeled phalloidin (A12379, Invitrogen) at a concentration of 1:200. After immunofluorescence staining, protein expression was observed with a fluorescence microscope (Ti-U, Nikon).

### ELISA Assays

For detecting the release of interleukin 6 (IL-6), the human IL-6 ELISA kit from Invitrogen (Thermo Fisher Scientific) was utilized. The supernatant of the plate cell culture wells was aspirated after the set exposure time to the treatments. The supernatant of the microfluidic chips was aspirated out of the medium. The test was carried out by following the protocol.

### Statistical Analysis

The data were analyzed via Student’s *t*-test or one-way ANOVA using the GraphPad Prism software and expressed as the mean ± standard deviation based on observations of the 10 representative fluorescent images obtained from three independent experiments. *P*-values <0.05 were considered statistically significant.

## Results and Discussion

### Fabrication of the Magnetic Driven Chip

**FIGURE 1:**
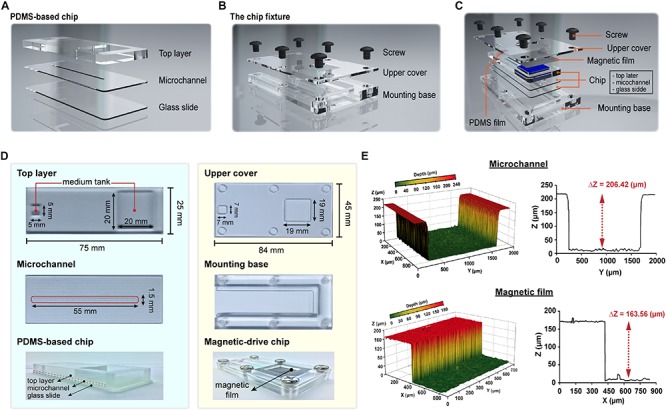
Design and fabrication of the magnetic driven chip. **(A)** Schematic of PDMS-based three-layer chip containing top layer, microchannel, and glass slide. **(B)** Schematic of chip fixture containing upper cover and mounting base. **(C)** Designing an assembly drawing of magnetic driven chip and its components (PDMS-based chip, chip fixture, and PDMS/magnetic film). **(D)** Fabrication of a magnetic driven chip includes a chip with process combining PDMS and glass slide (left side) and chip fixture of transparent acrylic plastic plates (right side). **(E)** The 3D surface morphology measurement of microchannels and magnetic films. *Z*-axis depth analysis showed that the height of the chip microchannel was 206.42 μm and the film thickness was 163.56 μm.

The overall structure of the proposed magnetic driven chip can be primarily divided into two parts – (i) a PDMS-based three-layer chip comprising a top layer, middle layer (microchannel), and glass slide (bottom layer) (Figure 1A and Supplementary Figure S1) and (ii) a chip fixture comprising an upper cover and mounting base (Figure 1B). The layers in the first part are bonded to each other by oxygen plasma activated bonding. The upper layer of the chip is a rectangular cuboid contains two square slots. These empty slots are primarily used to hold the cell medium. The second layer is a microchannel layer while the third layer is a glass slide. After the second and third layers are bonded, a microchannel is formed to constitute the area for cell culture. Finally, the magnetic and PDMS films are placed on the cell-culture-medium container slot formed on the chip, respectively. The upper cover and mounting base of the second part both contain six M6 holes (a hole with diameter measuring 6 mm and containing a thread) (Figure 1C). In addition, two square areas are carved of the upper cover. The area inside the fixture is responsible for fabricating the said three-layer chip (Figure 1D). The thickness of the magnetic film and chip-microchannel height were measured to be 163.56 and 206.42 μm, respectively, using a 3-D shape measuring instrument (Figure 1E and Supplementary Figure S2). The chip and fixture were sealed via fixation using a 1.5 N.m torque screw, thereby completing the assembly of the entire magnetic driven chip system (Supplementary Video S1).

### Manipulation of the Magnetic Driven Chip

**FIGURE 2:**
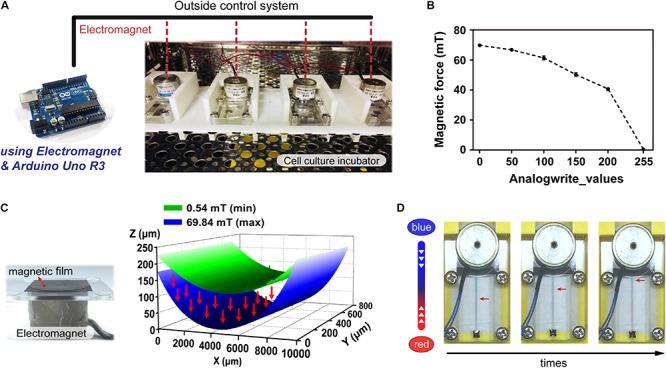
Operation and control of the magnetic driven chip. **(A)** Actual image of a magnetic driven chip used for cell culture. The electromagnetism (magnetic force) and elastically deformed magnetic film of magnetic driven chip inside an incubator at 37°C was controlled by an external Arduino control board. **(B)** Measurement and analysis of the change in magnetic force of the electromagnet. The magnetic field corresponding to different analogWrite() values recorded by the magnetic field Gauss meter in the Arduino program ranged from 0.54 to 69.84 mT (*n* = 5). **(C)** Deformation of the magnetic film after being subjected to different magnetic fields. The 3D surface morphology measurement results showed that the distance of magnetic film deformation corresponding to magnetic fields of 0.54–69.84 mT could reach 203.49 μm. **(D)** Status of the fluid during long-term fluid flow. Two liquids with different colors (red/blue) had no leakage and maintained a stable flow field.

To facilitate performing cell culture experiments on the proposed magnetic driven chip, the said magnetic chip was first set firmly along with an electromagnetic module inside a constant temperature incubator maintained at 37°C (Figure 2A). When the electromagnetic module was placed above the magnetic film, the film underwent different types of deformations depending on the magnetic force magnitude. The said magnitude could be used to control the electromagnetic module that transmits electrical signals to the incubator through a connecting wire using an Arduino control board (Supplementary Figure S3). To determine the limit of the magnetic force in this system, we divided the magnetic force into 0–255 equal parts, which were recorded using a magnetic field Gauss meter. The magnetic force can be sequentially controlled in PWM mode, with the analogWrite() function which analog value – 0, 50, 100, 200, and 255. For analogWrite() = 255, the magnetic force of the electromagnet was observed to be minimum, and its maximum values equaled approximately 69.84 mT for analogWrite() = 0 (Figure 2B). Figure 2C demonstrates that when the magnetic force equals 69.84 mT, the film-shape variable measures 203.49 μm. This serves to effectively drive the liquid through flow channels, thereby resulting in dynamic flow. Figure 2D demonstrates that upon injection of two colored dyes into the flow, the system continues to operate stably for a considerable duration without occurrence of any dye leakage. As can be observed in the figure, driving the magnetic film using a magnet makes the red-colored dye flow onto the other side owing to difference in pressure. Flow of the dye on the chip demonstrates feasibility of the magnetically driven chip system to sustain stable dynamic flow (Supplementary Video S2).

### Programmable Control of the Magnetic Driven Chip

**FIGURE 3:**
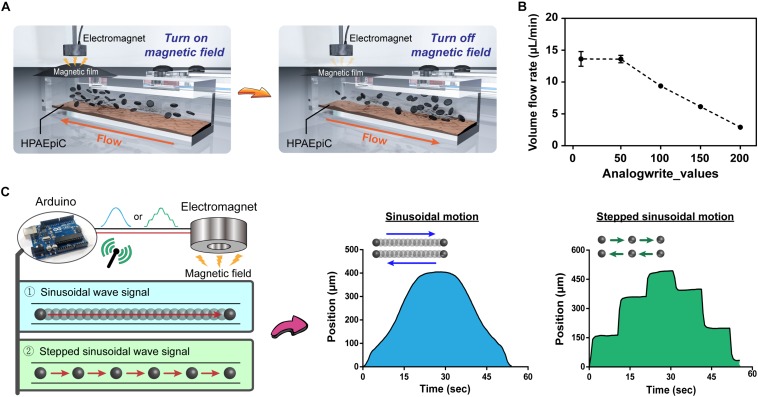
Programmable control of fluid flow patterns and rates. **(A)** Schematic of immediate control of changes in the magnetic film. The chip system does not contain any microfluidic tubing device, and the flow of liquid can be rapidly and easily changed through programmed changes in the magnetic field using Arduino. **(B)** Fluid flow rates corresponding to the magnitude of the magnetic field. Adding nanoparticles to the liquid allowed for visual observation and calculation through microscopy, with flow rates ranging from 2.90 to 13.64 μL/min (*n* = 3). **(C)** Evaluation of changes in magnetic fields by different signal patterns, sinusoidal and stepped sinusoidal signals were used for cyclical flow in the microchannel, and the non-linear flow rate (non-fixed value) could be adjusted flexibly to meet various conditions in the body.

An important advantage of the proposed system is that it does not require use of complicated structures, such as pumps and tubes, and that it is simple to implement. Arduino controls the magnetic force-induced deformation of the magnetic film, thereby establishing a simple and controllable fluid dynamic environment (Figure 3A). To further elucidate the feasibility of controlling the flow rate within a microchannel, nanoparticles were introduced into the flowing liquid, and the flow velocity of particles within the channel was calculated using the tracker video analysis and modeling tool (see text footnote 1). Subsequently, the liquid flow rate (μm/s) was converted to volumetric flow rate (μL/min) (Supplementary Table S1). Figure 3B demonstrates that for analogWrite() values of 0, 50, 100, 150, and 200, the corresponding volumetric flow rates equal 18.18, 18.14, 12.53, 8.18, and 3.88 μL/min, respectively, thereby indicating that the magnetically driven chip system can not only drive liquid flow but also dynamically change the flow rate within channels in real time by making adjustments to the magnetic force and magnetic film deformation parameter. In addition, the Arduino program can be used to control the magnetic force by using different signal output methods, such as sinusoidal and stepped sinusoidal signals to control the cyclic flow of liquid within flow channels (Figure 3C and Supplementary Videos S3, S4). Compared to straight line linear flow systems, the fluid demonstrates not only a fixed but also an adjustable flow pattern, thereby provides new options for performing microfluidic cell cultures.

### Magnetic Driven Chip for Modeling the Alveolar

**FIGURE 4:**
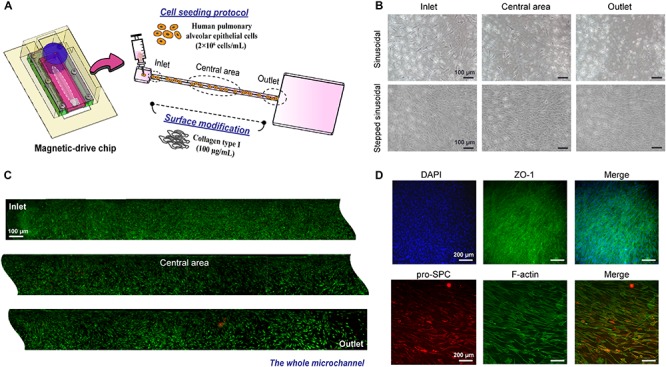
HPAEpiC cells cultured in a dynamic magnetic driven chip. **(A)** Schematic of cell seeding in the chip. First, the substrate was treated with collagen, and cell suspension with a concentration of 2 × 10^6^ cells/mL was injected into the microchannel. **(B)** Effect of magnetic field signaling mode on cell growth. Cells were cultured for 5 days using the sinusoidal signal and stepped sinusoidal signal, and the stepped sinusoidal had the optimal cell density and morphology. Stepped sinusoidal signals were thus used in subsequent experiments. Scale bar is 100 μm. **(C)** Fluorescence of viable cells cultured in a dynamic flow environment. After 7 days of continuous perfusion flow field driven by the magnetic film, it was observed by LIVE/DEAD assay that the entire flow channel exhibited good cell viability and sufficient growth. Scale bar is 100 μm. **(D)** The functional characterization of cells cultured in a dynamic flow environment. Immunofluorescence image of a monolayer formed by HPAEpiC cells after 7 days of culture, which included a barrier function (tight junction protein 1, ZO-1) and specific pro-surfactant protein C (pro-SPC) secreted by HPAEpiC. Scale bar is 200 μm.

To further understand advantages of the proposed method for use in cell culture and future tissue chip applications, HPAEpiCs were cultured using the proposed magnetically driven dynamic system with collagen coating being used as the chip–substrate interface material (Figure 4A). The cell concentration inoculated on the chip was set as 2 × 10^6^ cells/mL (Supplementary Figure S4), and the effects of liquid circulation on cell culture were observed using different output signals, such as sinusoidal and stepped sinusoidal signals. Results of this investigation demonstrated the cells to be in their best condition after 5 days of cell proliferation when fluid flow was controlled using a stepped sinusoidal signal (Figure 4B). Additionally, the shear stress and nutrient exchange functions realized by the flow rate were deemed to be most suitable for cell culture. Figure 4C demonstrates that observation of the cells via LIVE/DEAD assay revealed no obvious cell death in the chip after 7 days of dynamic culture. Recapitulation of the effect on cell viability within channels revealed the cells to remain intact and uniform when cultured using the proposed on-chip system. Fluorescence staining facilitated clear observation of the tight-junction marker zonula occludens-1 (ZO-1), produced by tight junctions of the cell in real time, along with the specific surfactant protein C (SPC) secreted by AEII cells (Figure 4D). This implies that the proposed culture method not only leads to uniform cell culture on-chip but also maintains the barrier function and differentiation ability of AEII cells. In view of these advantages, microchannel cell culture, such as that performed on cell or organ chips, is no longer dependent on only a single parameter for flow rate control, and that use of a magnetic drive effectively controls the fluid flow pattern in the step-signal-output mode. The frequency and shear stresses generated within the chip tend to rapidly adjust optimum growth conditions for the cells (Supplementary Table S2), thereby recapitulating the cell structure and maintaining the cell-differentiation ability. This helps us realize the advantages of *in vitro* analysis of the tissue function and cell-biochemical activity.

### Functionality of the Magnetic Driven Alveolus Cell-Culture Chip

**FIGURE 5:**
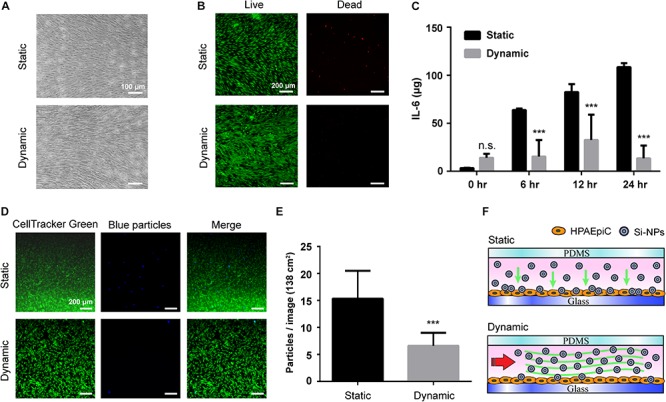
Magnetic driven chip applied to inflammatory reactions to stimuli. **(A)** Bright field images of cells cultured on static and dynamic environment after exposure to Si-NPs at a concentration of 50 μg/mL for 24 h. Scale bar is 100 μm. **(B)** After 24 h treatment of Si-NPs, the cells were observed by LIVE/DEAD assay staining that cell viability in the static culture was significantly lower than in the dynamic culture. Scale bar is 200 μm. **(C)** Comparison of the immune response after Si-NPs stimulation. The concentration of IL-6 produced by cells in the static environment was much higher, and the concentration increased as the time of exposure increased (^∗∗∗^ *p* < 0.001; *n* = 3). **(D)** Fluorescence of particles deposited in dynamic and static environments. After adding blue fluorescent particles (blue particles), the number of particles deposited in the cells in the static environment after 24 h was significantly higher than in the dynamic environment. Scale bar is 200 μm. **(E)** Quantification of the number of blue particles deposited on the cells. The particles deposited on the cells were calculated from fluorescent signals, and the results showed that the number in the static environment was twice that in the dynamic environment (^∗∗∗^*p* < 0.001; *n* = 10). **(F)** Schematic of the particle deposition process when exposing Si-NPs that is added in the magnetic driven chip with static and dynamic environment.

To thoroughly compare the differences in cellular response between the proposed magnetic driven system and generic static culture methods, a stimulation was performed using silica nanoparticles (Si-NPs) at a concentration of 50 μg/mL and identical cell density (Figure 5A). Figure 5B demonstrates that cells grown using static culture methods exhibit considerable cell death after 24 h of stimulation, whereas minimal cell death can be in cells grown using the magnetic driven system. Figure 5C illustrates a significant difference in the IL-6 inflammatory response of Si-NPs between static and dynamic systems. The IL-6 concentration expressed after performing the Si-NPs stimulation in a static environment was observed to be much higher with a tendency to increase with increase in stimulation time. A similar phenomenon was observed when comparing results obtained through use of a conventional peristaltic pump with static cells. Silica-treated cells in the dynamic system were, in general, observed to be healthy with a few dead cells spreading over the cell complex (Rothbauer et al., 2015; Yazdimamaghani et al., 2018). Thus, regarding to device simplification, use of the proposed magnetic driven system not only provides advantages but also facilitates on-chip fluid flow to be controlled using the magnetic-output mode, which in turn facilitates rapid attainment of optimum growth conditions for cells in a feasible manner. Comparing the results of cytotoxicity and immune reactions obtained via Si-NPs stimulation also demonstrates that use of the proposed system results in occurrence of a smaller number of inflammatory reactions compared to the static cell culture system. This result is similar to that obtained using a dynamic system driven by a conventional peristaltic pump (Supplementary Figure S5). In order to further investigate the difference in reactions stimulated by Si-NPs between the dynamic and static cell-culture environments, cell cultures were performed using blue fluorescent polystyrene particles (blue particles) in identical concentrations for 24 h in using either the magnetic driven system or static culture environment (Figures 5D,E). Corresponding results demonstrated that compared to the static environment, polystyrene particles were less likely to adhere to cells in the dynamic environment (Rothbauer et al., 2015). Thus, cell deaths and inflammatory reactions caused by the particles may not be significant (Figure 5F). This result is also consistent with dynamic environment phenomena occurring inside the human body (Björnmalm et al., 2016). In addition to demonstrating the feasibility of using a magnetic driven system for cell culture and stimulation within a dynamic environment, these results also demonstrate that magnetic driven systems mimics the dynamic environment of the human body more closely, thereby generating a relatively greater amount of meaningful data.

## Conclusion

The present study describes the development of a new type of magnetically driven chip. The proposed system facilitates selection of magnetically controllable output modes, and employs a magnetic film as a medium to control fluid circulation through on-chip microchannels, thereby overcoming bottlenecks associated with primary on-chip human cell culture while also addressing problems pertaining to uniform and long-term on-chip culture of alveolar cells along with recapitulation of the cell structure and cell differentiation functions (Supplementary Figure S6). Reactions between human alveolar epithelial cells and Si-NPs were assessed to validate differences between the dynamic environment produced by the chip and conventional static environments, thereby realizing functions and advantages of *in vitro* tissue construction. Numerous techniques have been developed in recent years to construct organs and/or tissues on chips to resolve the enormous differences between animal experiments and clinical trials (Capulli et al., 2014; Ahadian et al., 2018).

However, representing individualized differences on chips yet remains a major challenge. The proposed system does not only not require complicated structures, such as pumps and tubes, thereby simplifying device construction, but also greatly reduces external factors derived from equipment requirements and human influenced factors during experiments. The system also possesses a wireless connection system capable of collecting breathing signals of individuals. It can simulate the most realistic individualized breathing models by converting parameters, such as individual respiratory rate and respiratory force. This helps facilitate collection of more realistic and effective information with regard to drug screening as well as establishment of individualized disease models. In view of these advantages, the system demonstrates great potential for use in applications related to bionic engineering of tissue and organ chips.

## Data Availability Statement

All datasets generated for this study are included in the article/Supplementary Material.

## Author Contributions

J-WY, Y-WC, and G-YC contributed to the conception of the study. J-WY, Y-WC, P-YH, and LJ carried out the experiments. KH, S-JC, and K-CL helped perform the analysis with constructive discussions. H-EL, H-YC, S-FL, and G-YC supervised the research design. J-WY and G-YC wrote the manuscript. All authors listed have made a substantial, direct and intellectual contribution to the work, and approved it for publication.

## Conflict of Interest

The authors declare that the research was conducted in the absence of any commercial or financial relationships that could be construed as a potential conflict of interest.

## Footnotes

1 https://physlets.org/tracker/
