# Supplementary material for: The Development of Controllable Magnetic Driven Microphysiological System
Source: Front Cell Dev Biol. 2019 Nov 7;7:275. doi: 10.3389/fcell.2019.00275 (PMC6853840; doi:10.3389/fcell.2019.00275)
Supplement: Supplementary file 1 [file Data_Sheet_1.doc]

**Supporting Information**

**The Development of Controllable Magnetic Driven Microphysiological System**

Jia-Wei Yang^1,2^ , Yu-Wei Chen^2^ , Pei-Yi Ho^2^ , Liane Jiang^2,3^ , Kuan Yu Hsieh^1,2^, , Sheng-Jen Cheng ^1,2^ , Ko-Chih Lin^1,2^ , Huai-En Lu^4^ , Hsien-Yi Chiu^5,6,7^ , Shien-Fong Lin^1,2^ and Guan-Yu Chen^2,8*^

^1^ Department of Electrical and Computer Engineering, College of Electrical and Computer Engineering National Chiao Tung University, Hsinchu, Taiwan

^2^ Institute of Biomedical Engineering, College of Electrical and Computer Engineering, National Chiao Tung University, Hsinchu, Taiwan

^3^ Section for Experimental Ophthalmic Surgery and Refractive Surgery, Centre for Ophthalmology, University of Stuttgart, Germany

^4^ Bioresource Collection and Research Center, Food Industry Research and Development Institute, Hsinchu, Taiwan

^5^ Department of Dermatology, National Taiwan University Hospital Hsin-Chu Branch, Hsinchu, Taiwan

^6^ Department of Dermatology, College of Medicine, National Taiwan University, Taipei, Taiwan

^7^ Department of Dermatology, National Taiwan University Hospital, Taipei, Taiwan

^8^ Department of Biological Science and Technology, National Chiao Tung University, Hsinchu, Taiwan

*Corresponding author

Phone: (886) 3-573-1920

FAX: (886) 3-573-1672

Email: guanyu@nctu.edu.tw

**Keywords:** Magnetic driven, Microphysiological systems, Human alveolar epithelial cells, Barrier function, Silica nanoparticles


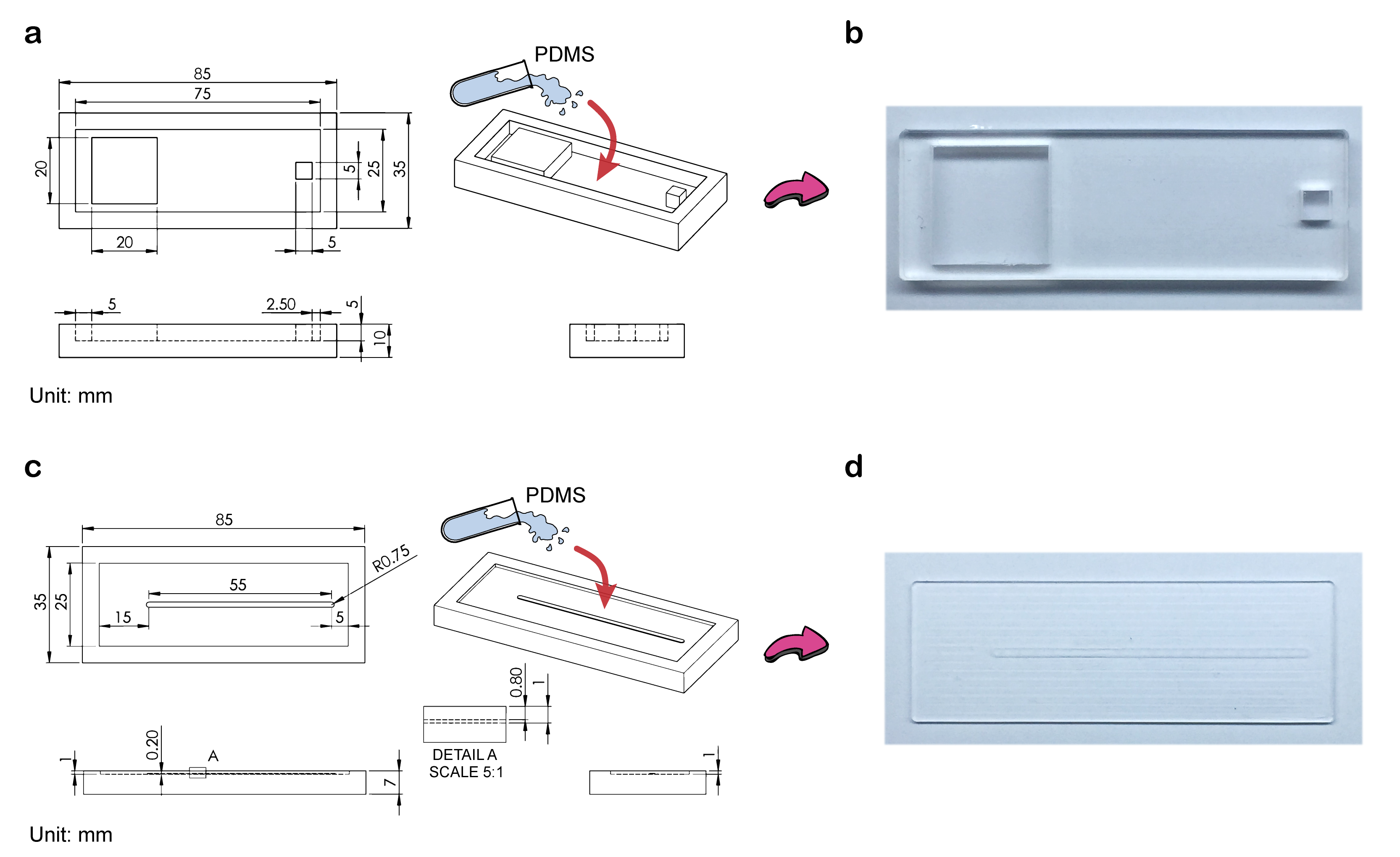


**Figure S1.** Preparation and characterization of the PDMS-based three-layer chip. (a) Mechanical drawing of the top layer mold. The molds are made of acrylic and then poured mixed PDMS into the mold to form a product by heat curing. (b) Photographs of the top layer of PDMS-based three-layer chip. The structure contains two cell culture medium loading spaces. (c) Mechanical drawing of the middle layer (microchannel) mold. (d) Photographs of the microchannel layer of PDMS-based three-layer chip. The structure contains an elongated cell culture space (55 mm (L) × 1.5 mm (W) × 0.2 mm (H)).

**
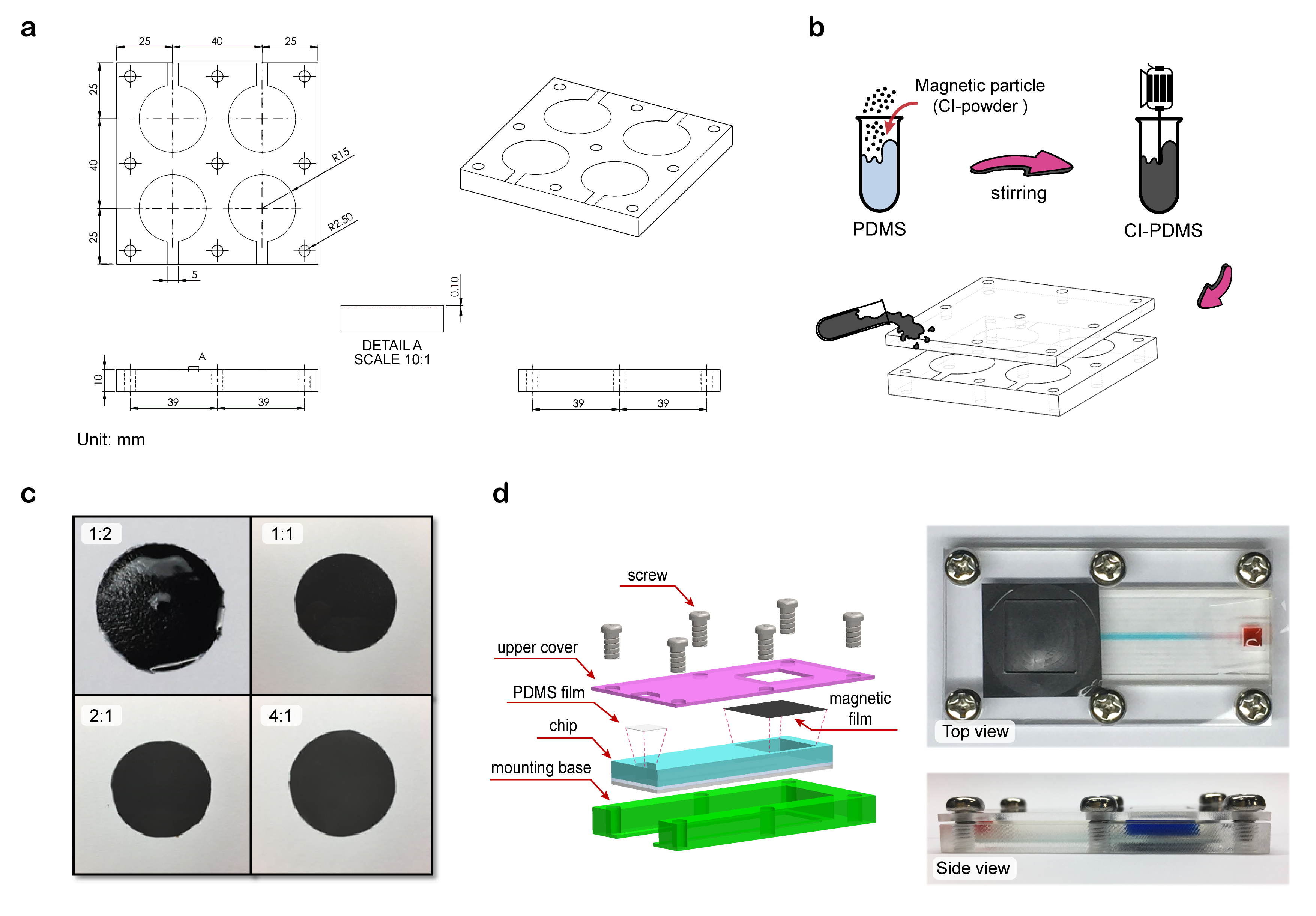
**

**Figure S2.** Preparation and characterization of the magnetic film. (a) Mechanical drawing of the magnetic film mold. The molds are made of acrylic and the structure contains four film forming spaces. (b) Preparation and manufacture of magnetic films. The magnetic mixture(CI-PDMS) consists of CI-powder and PDMS as a magnetic film material with elastic properties. (c) Photographs of various magnetic films with different CI and PDMS ratios. After evaluating elasticity and magnetic force, we used a CI-PDMS mixture in a weight ratio of 2:1 as experimental conditions. (d) Schematic diagram of magnetic film and magnetic drive chip assembly. The magnetic film is fixed by screws between the PDMS-based three-layer chip and chip fixture.

**
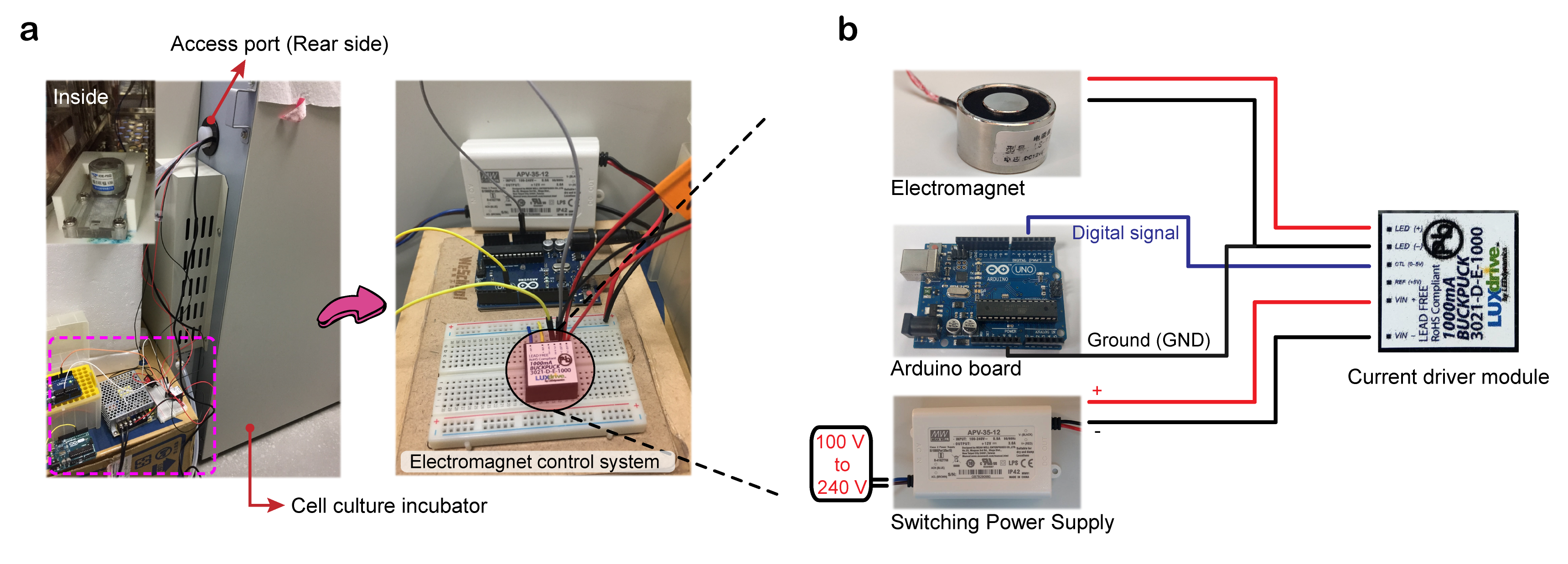
**

**Figure S3.** Demonstrating a simple electromagnet controlled through an Arduino board and current LED driver module. (a) Actual image of an electromagnet control system placed outside the cell incubator. Connect the internal electromagnet to the external control system through the access port on the rear of the incubator. (b) Schematic diagram of the electromagnet control system and its components. The overall setup configuration includes electromagnet, Arduino UNO board, switching power supply, and current driver module.

**
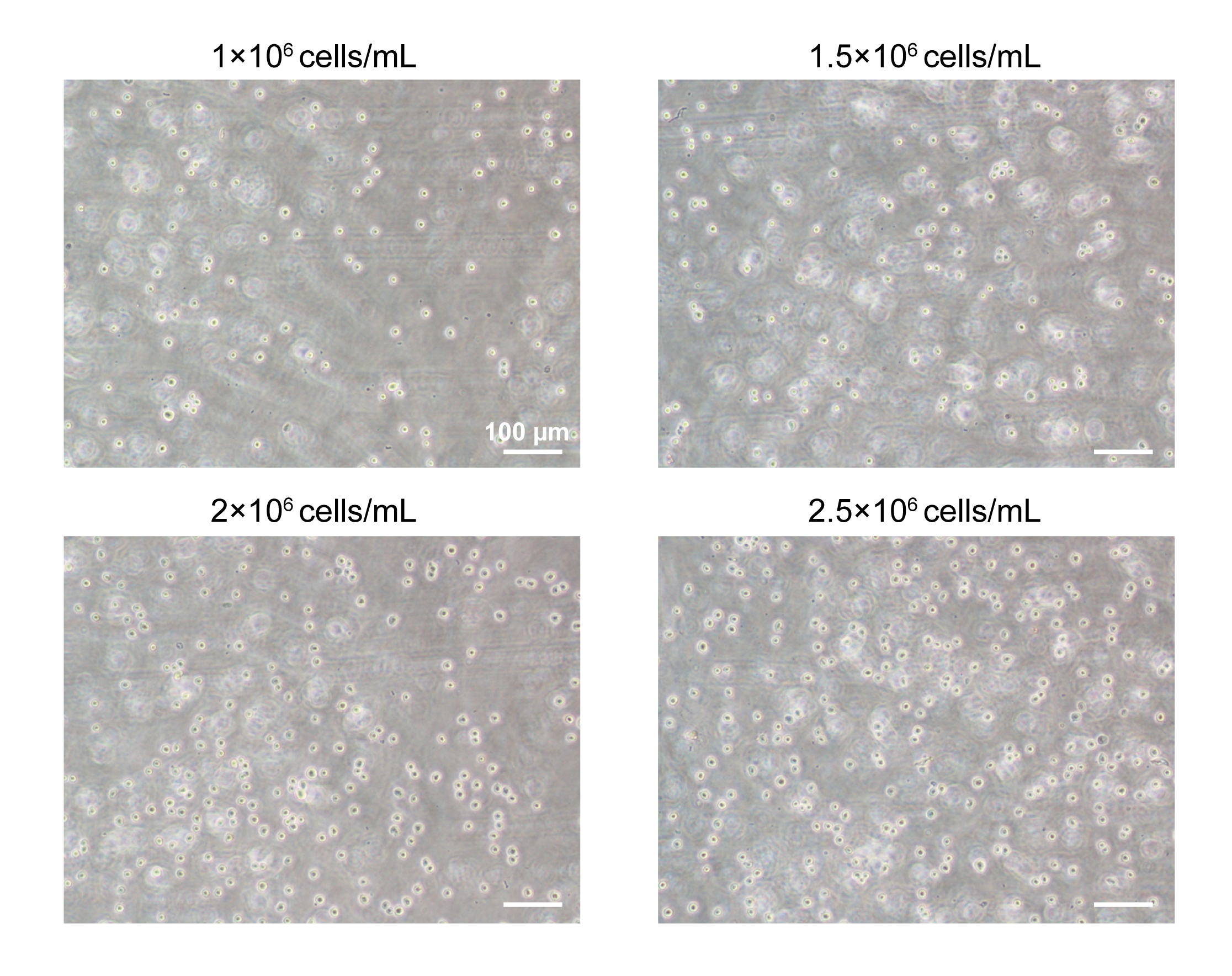
**

**Figure S4.** Assessing the appropriate HPAEpiC cells seeding density in the microchannel of the chip. As seen in picture, the cells concentration increased from 1×10^6^ cells/mL to 2.5×10^6^ cells/mL, the density of HPAEpiC cells increases with increasing concentration, determining the optimal cell concentration of at 2×10^6^ cells/mL for selected condition. Scale bar is 100 μm.

**
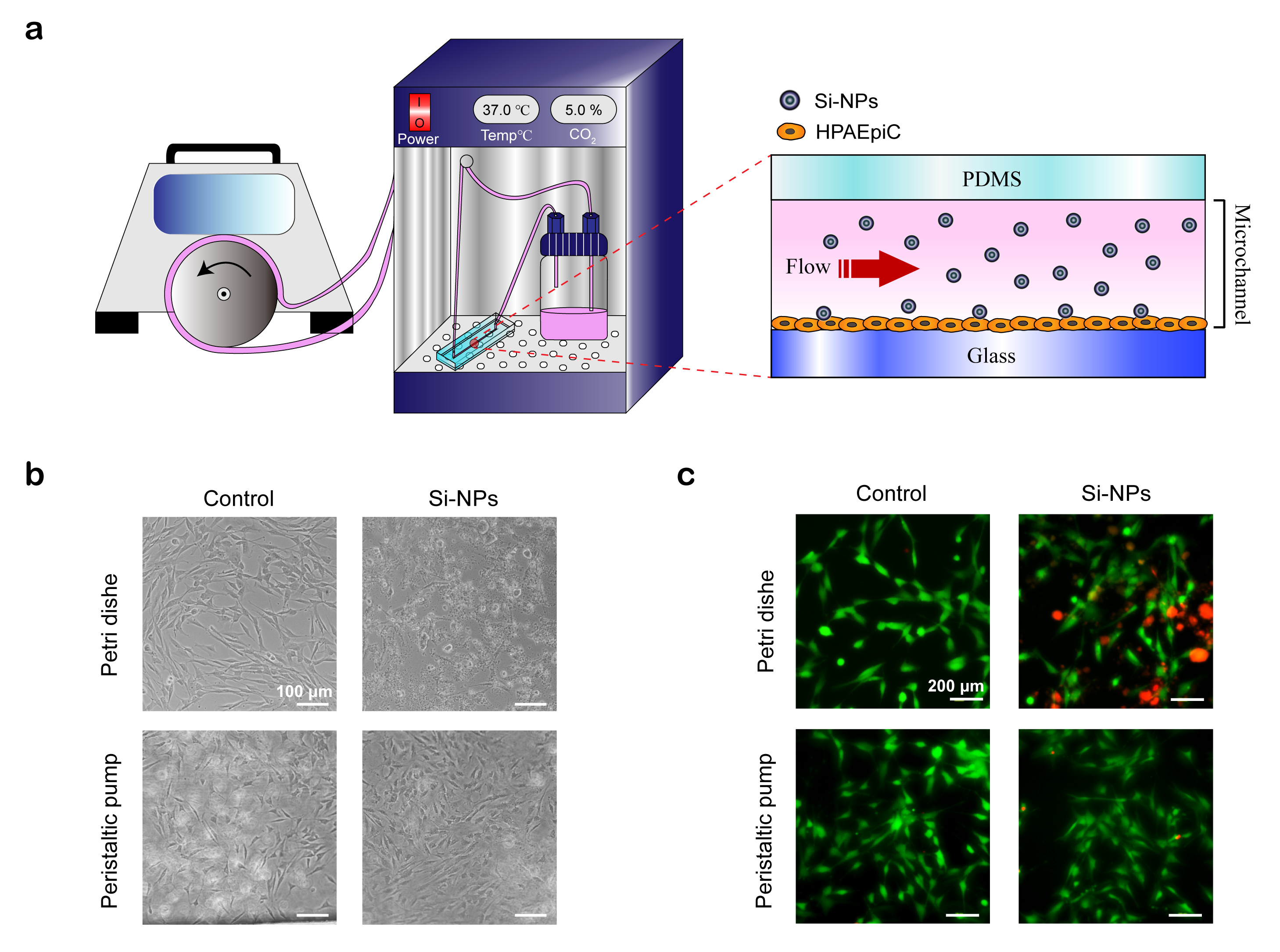
**

**Figure S5.** HPAEpiC cells perfusion cultured in the chip using a peristaltic pump. (a) The working principle of dynamic perfusion, the close loop system were formed after connecting each side of chip to bottle of fresh medium and peristaltic pump, then the culture medium were driven by pump to create a dynamic environment. (b) Bright field images of cells cultured on petri dish and dynamic perfusion environment within 6 hours after Si-NPs treatment.The cells break up into small pieces in static environment, but were not observed in the dynamic perfusion environment. Scale bar is 100 μm. (c) Live/dead viability staining of HPAEpiC cells after 6 hours Si-NPs treatment. The cells were dual-color stained assay to discriminate the live (green) and dead cells (red); however, the results showed higher stimulation and caused a lot of death in static environment. Scale bar is 200 μm.

**
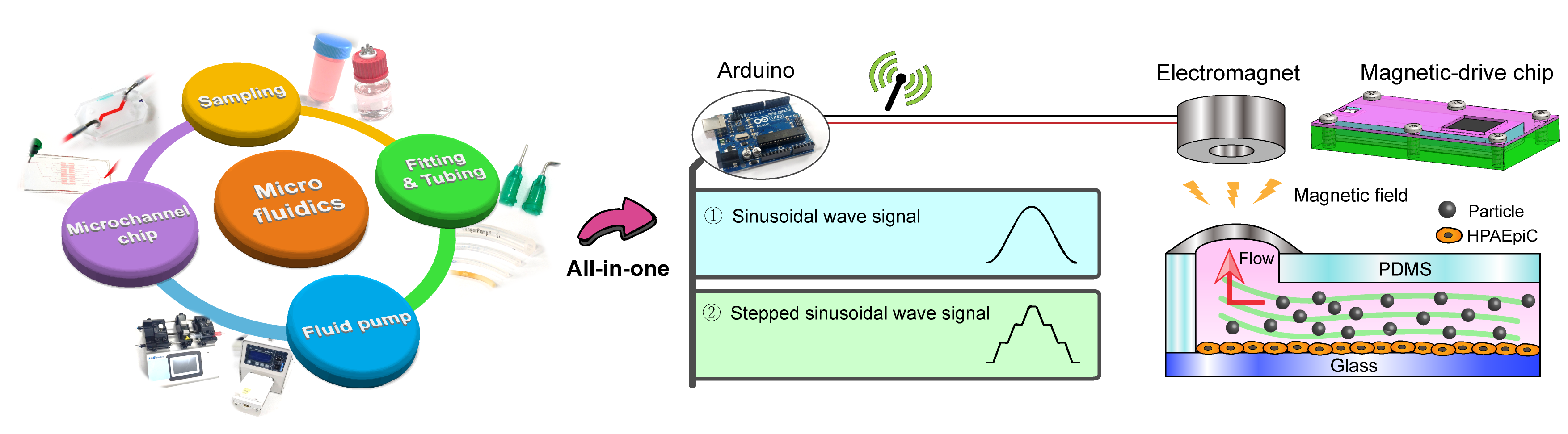
**

**Figure S6.** Simple and controllable magnetic driven microfluidic system. Schematic of common microfluidic operations including microchannel chip, liquid sample, delivery power sources (pumps), and tubing. Complex and multi-step connections are simplified and integrated into the magnetic driven chip that reduces fluid flow instability, bubble and leakage problems.

**Table S1.** The flow velocity and volume flow rate in magnetic driven chip

| analogWrite() values | | Flow velocity (µm/sec) | | Cross-sectional area of the moicrochannel (mm^2^) | | Volume flow rate^*^ (µL/min) |
| --- | --- | --- | --- | --- | --- | --- |
| 0 | 757.60±57.33 | | 0.3 | | 13.64±1.03 | |
| 50 | 756.00±28.97 | | 0.3 | | 13.61±0.52 | |
| 100 | 522.16±11.16 | | 0.3 | | 9.40±0.20 | |
| 150 | 340.97±15.69 | | 0.3 | | 6.14±0.28 | |
| 200 | 161.54±4.97 | | 0.3 | | 2.90±0.90 | |

^*^ The volume flow rate equation used by formula is *Q* = *A* · *v* , where *Q* is the volume flow rate, *A* is the cross-sectional area of the moicrochannel (1.5 mm × 0.2 mm), and *v* is the flow velocity.

Table S2. The flow reynolds number and shear stress in the magnetic driven chip

| analogWrite() values | Reynolds number^#^  (Re) | Fluid shear stress% (dyn/cm2) |
| --- | --- | --- |
| 0 | 37.04±2.80 | 0.16±0.01 |
| 50 | 36.96±1.42 | 0.16±0.01 |
| 100 | 25.53±0.55 | 0.11±0.00 |
| 150 | 16.67±0.77 | 0.07±0.00 |
| 200 | 7.90±0.24 | 0.03±0.00 |

^#^ The reynolds number (Re) equation used by formula is *Re* = *ρVL/µ* .

% The fluid shear stress (τ) equation used by formula is *τ = 6µQ/bh^2^* .

Here, *ρ* is the medium density (0.9973 g/mL), *V* is the fluid velocity, *L* is the characteristic length (for the rectangular microchannel can be equal to 2*bh/b+h*)*, µ* is the dynamic viscosity of medium (0.0072 dyn·s/cm² from Shear Stress and Shear Rates for ibidi μ-Slides-Based on Numerical Calculations, ibidi GmbH), *Q* is the volume flow rate, *b* is the width of microchannel, and *h* is the height of microchannel.

**
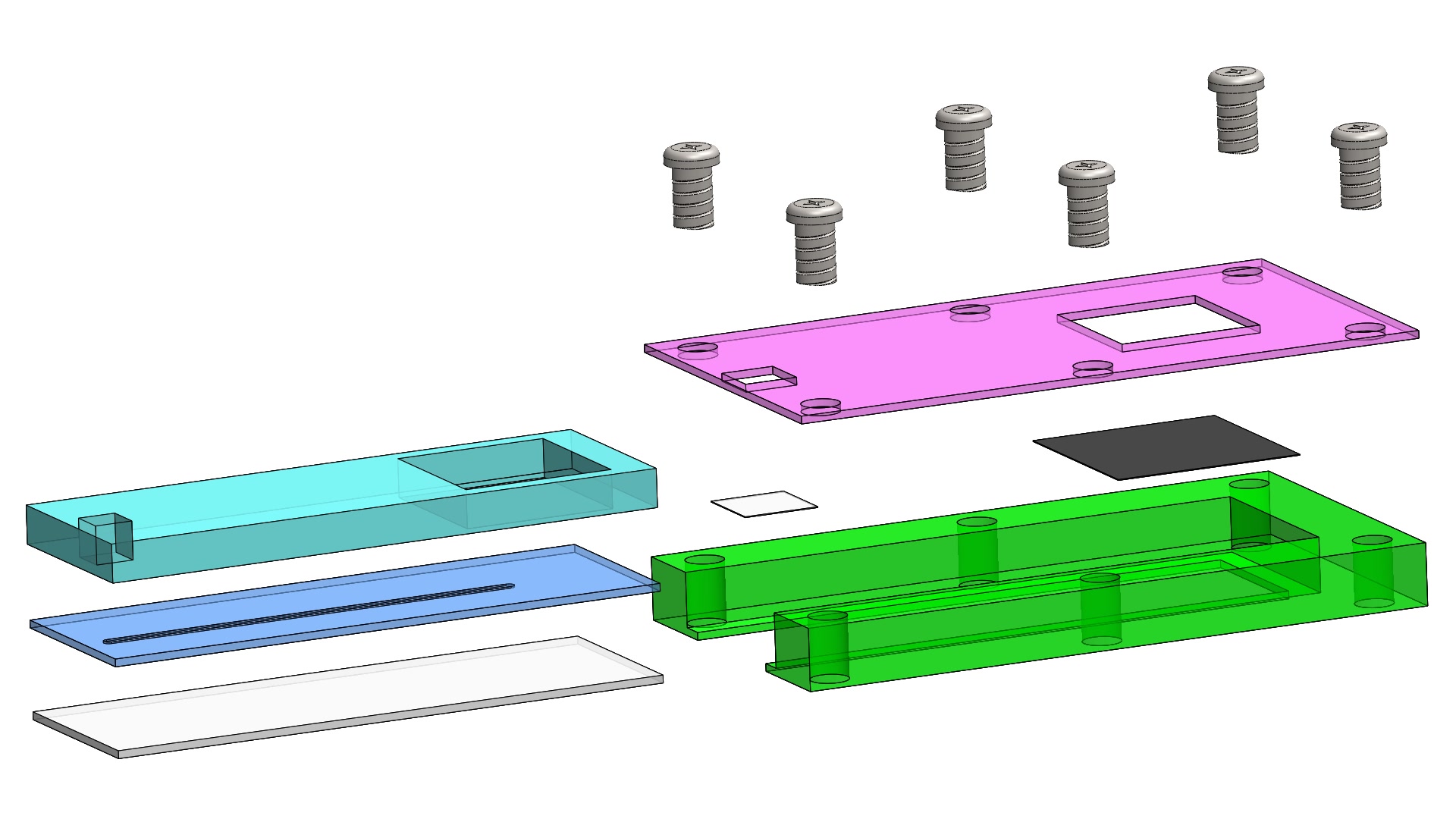
**

**Video S1.** The assembly of the magnetic driven chip includes a PDMS-based three-layer chip and chip fixture.


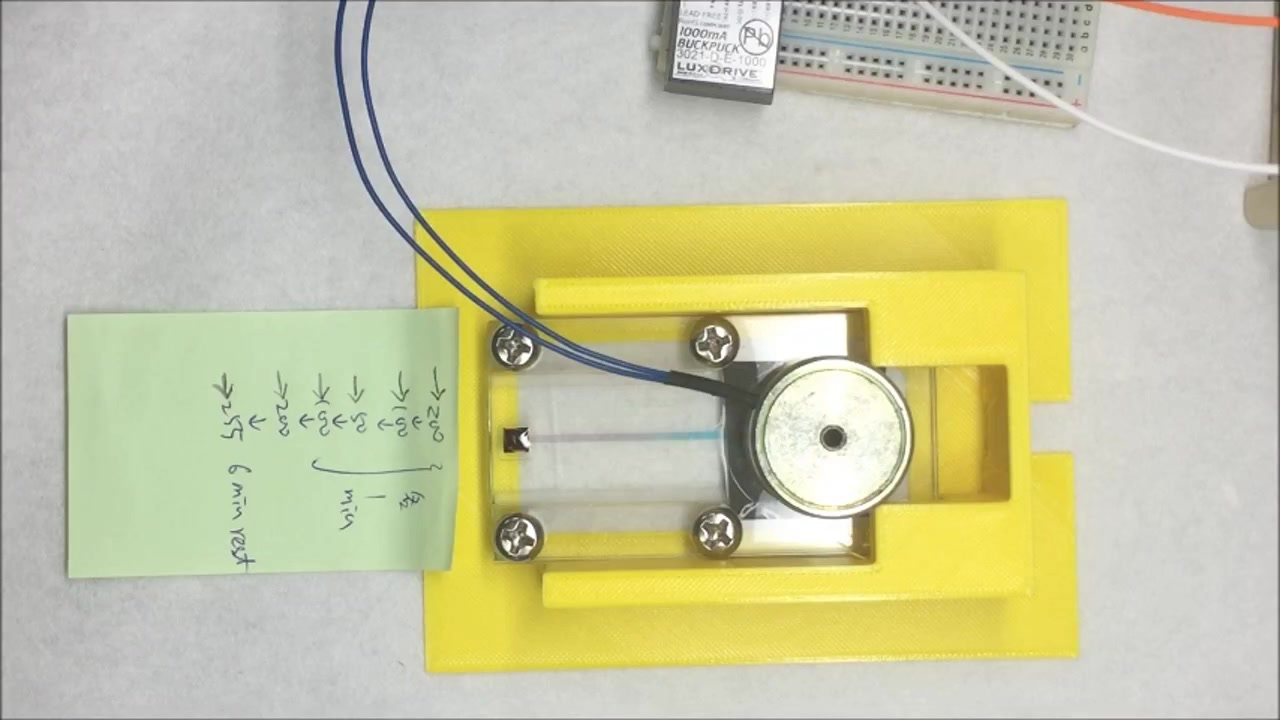


**Video S2.** Evaluating the dynamic environment of a magnetic driven chip through color-flow.


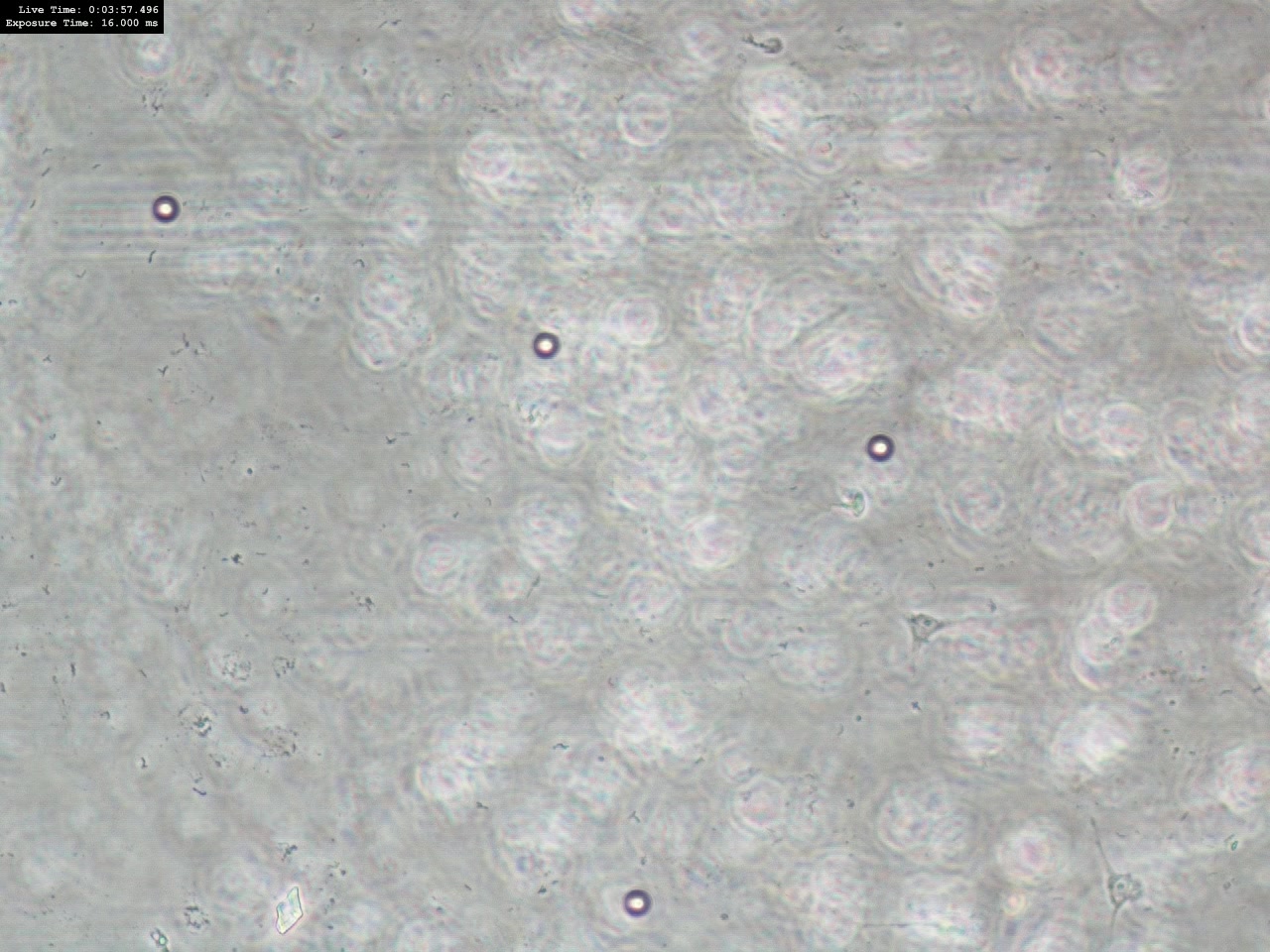


**Video S3.** Supplementary movie for the particles flow in the sinusoidal signal magnetic field.


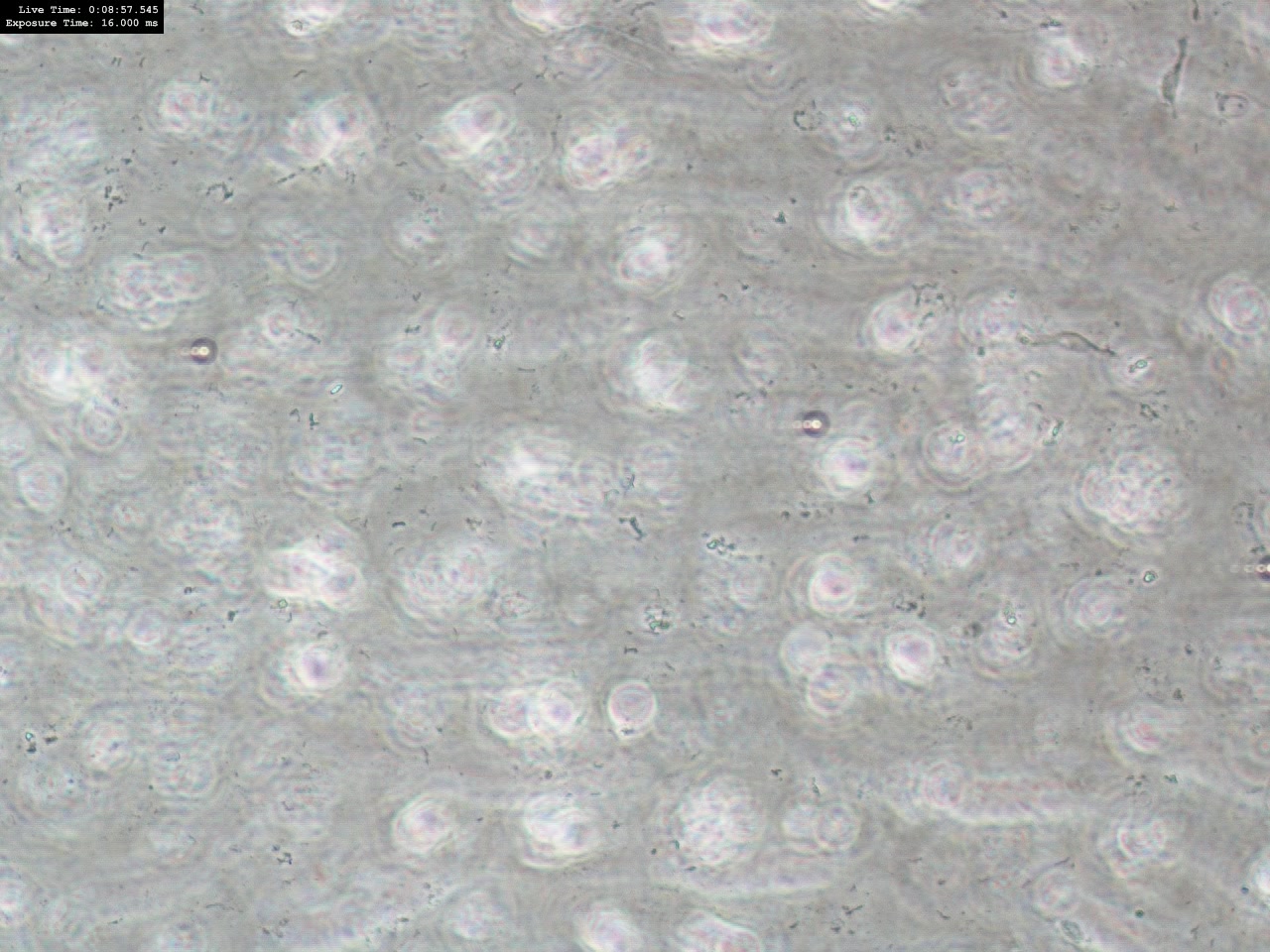


**Video S4.** Supplementary movie for the particles flow in the stepped sinusoidal signal magnetic field.

**References**

Hamilton, G.A., Jang, K.-J., McPartlin, L.A., Mehr, A.P., Chung, S., Ingber, D.E., Suh, K.-Y., 2013. Human kidney proximal tubule-on-a-chip for drug transport and nephrotoxicity assessment. Integr Biol 5(9), 1119-1129.

Toh, A.G.G., Wang, Z.P., Yang, C., Nguyen, N.-T.J.M., Nanofluidics, 2014. Engineering microfluidic concentration gradient generators for biological applications. Microfluid. Nanofluid. 16(1), 1-18.
